# 3D synchrotron imaging of muscle tissues at different atrophic stages in stroke and spinal cord injury: a proof-of-concept study

**DOI:** 10.1038/s41598-022-21741-z

**Published:** 2022-10-14

**Authors:** Jessica Pingel, Hans Martin Kjer, Fin Biering-Sørensen, Robert Feidenhans’l, Tim B. Dyrby

**Affiliations:** 1 Department of Neuroscience, University of Copenhagen, Copenhagen, Denmark; 2 Department of Applied Mathematics and Computer Science, Technical University of Denmark, Lyngby, Denmark; 3 Section for Spinal Cord Injuries, Department for Brain and Spinal Cord Injuries, Rigshospitalet and Institute of Clinical Medicine, University of Copenhagen, Copenhagen, Denmark; 4 Niels Bohr Institute, University of Copenhagen, Copenhagen, Denmark; 5 European X-Ray Free Electron Laser, Schenefeld, Germany; 6 Danish Research Centre for Magnetic Resonance, Copenhagen University Hospital Hvidovre and Amager, Hvidovre, Denmark

**Keywords:** Biophysics, Computational biology and bioinformatics, Neuroscience, Physiology, Medical research, Neurology, Engineering, Mathematics and computing

## Abstract

Synchrotron X-ray computed tomography (SXCT) allows 3D imaging of tissue with a very large field of view and an excellent micron resolution and enables the investigation of muscle fiber atrophy in 3D. The study aimed to explore the 3D micro-architecture of healthy skeletal muscle fibers and muscle fibers at different stages of atrophy (stroke sample = muscle atrophy; spinal cord injury (SCI) sample = severe muscle atrophy). Three muscle samples: a healthy control sample; a stroke sample (atrophic sample), and an SCI sample (severe atrophic sample) were imaged using SXCT, and muscle fiber populations were segmented and quantified for microarchitecture and morphology differences. The volume fraction of muscle fibers was 74.7%, 70.2%, and 35.3% in the healthy, stroke (atrophic), and SCI (severe atrophic) muscle fiber population samples respectively. In the SCI (severe atrophic sample), 3D image analysis revealed fiber splitting and fiber swelling. In the stroke sample (atrophic sample) muscle fiber buckling was observed but was only visible in the 3D analysis. 3D muscle fiber population analysis revealed new insights into the different stages of muscle fiber atrophy not to be observed nor quantified with a 2D histological analysis including fiber buckling, loss of fibers and fiber splitting.

## Introduction

A synchrotron X-ray computed tomography (SXCT) generates 3D images of tissue at micrometer image resolution with a large field-of-view (FOV)^1^ that covers typical muscle biopsies (3–5 mm cubes). A high photon flux, monochromatic, tunable beams, and the use of phase-contrast imaging can provide detailed tissue contrast not possible with classical absorption-based x-ray imaging^2^. As opposed to classical histopathological examinations based on 2D slices^1^, SXCT is a volumetric non-destructive imaging technique. It enables 3D insights into muscle fiber morphometry and architectural changes in pathophysiological processes such as muscle fiber atrophy and muscle fiber death in a whole new scale^3^. Skeletal muscle fibers have a unique capacity to adjust their metabolism and phenotype, not only in response to pathology, but also in response to alternations in mechanical loading. While chronic mechanical loading leads to an increase in skeletal muscle mass (hypertrophy), prolonged mechanical unloading and/or immobilization causes a significant decrease in muscle mass (atrophy)^4^. Long-term immobilization (6–10 weeks) with a short leg cast (ROM-walker) decreased the muscle mass of the triceps surae by 15pct. in patients with unilateral malleolus fractures^5^. Moreover, the standard remobilization protocol for the same period showed a recovery (increase) of the muscle mass by 9%^5^. Even though the recovery of the muscle is slow, the muscle loss caused by immobilization is fully reversible and can be accelerated by strength training^6^. A 2D histology analysis has previously revealed muscle atrophy and fiber type switching from slow to fast fiber types^7^. Whether such a muscle atrophy causes changes in muscle fiber morphology and microarchitecture is unclear. As opposed to long-term immobilization, denervation affects the muscle tissue more. While the muscle tissue affected by short-term denervation recovers well, the muscle tissue suffering from long-term denervation does not recover very well^8–10^. Thus, the recovery process from unilateral nerve injury induced in the hind limb of rats has been observed following nerve repair surgery shortly after the injury. However, if the nerve repair is done later than 4 weeks after the injury, the recovery is very poor^8,9^. Additionally, 4 weeks of denervation causes a muscle loss (atrophy) of 80–90% in the affected leg when compared to the unaffected side^11,12^. Functional electrical stimulation (FES) of skeletal muscle has shown promising effects regarding the prevention of muscle loss in individuals with spinal cord injury (SCI)^13–16^. However, even though these observations indicate a certain level of plasticity, the skeletal muscle tissue has its limits in regards to recovery after denervation and can reach a point where full recovery is no longer possible^9^. Previous studies have shown that individuals with SCI experience skeletal muscle fiber autophagy and cell death using 2D histology^17^. Furthermore, 2D histological analyses of skeletal muscle tissue from individuals with spinal cord injuries have shown fiber type transformation, contractile changes, and intramuscular fat and connective tissue accumulation^18,19^. However, how the muscle fiber morphology and microarchitecture on a muscle population basis are affected in 3D is still unclear.

Stroke may cause a unilateral paralysis of one arm and one leg, leaving the patient hemiplegic with an impaired gait but still ambulant^20^. In patients with SCI, the denervation is mostly bilateral and all muscles that are distal to their lesion may be paralyzed, causing the patient to become non-ambulant requiring a wheelchair^21^. Both injuries cause muscle atrophy and muscle weakness in the affected limbs. However, individuals with SCI often experience more prominent muscular symptoms than stroke survivors. Denervation has also been shown to cause a shift in muscle fiber type distribution^22^ and local inflammation in the muscle tissue^23^. Ultrasound studies have revealed that a stroke changes the muscle architecture, causes increased echogenicity and decreases muscle thickness^24^. However, current imaging techniques, including transmission electron microscopy, and confocal microscopy mostly limited to 2D images are measuring only the macroscopic architecture of skeletal muscles. Furthermore, these techniques can only track a single or few muscle fibers through the tissue. Whether the micro-architecture of the muscle changes at different levels of atrophy severity as seen after a stroke and SCI is still unclear. A 3D analysis of muscle tissue at different levels of atrophy might help to answer some of these questions.

In the present study, we aimed to elucidate differences in the 3D micro-architecture and morphology at different stages of a muscle fiber atrophy, using biopsies of three individuals: (1) A control muscle sample obtained from a healthy individual, (2) An atrophic muscle sample obtained from an individual who survived a stroke and was partly denervated on the affected side but still ambulant, and finally, (3) A severe atrophic sample obtained from an individual with an SCI (21 years after the injury) representing long-term denervation without ambulation. We hypothesized that the muscle atrophy that was caused by denervation might affect the 3D micro-architecture of the level on muscle fiber populations not seen with 2D histopathology and its changes with the degree of muscle atrophy severeness. The image analysis includes methods to segment and analyze muscle architecture as well as muscle morphology in 3D.

## Materials and methods

### Participants and biopsy samples

Before the experiment, the three individuals signed an informed consent form.Three muscle biopsies were taken: one biopsy from a healthy individual (referred to as a healthy control sample), one biopsy from an individual that had survived a stroke (referred to as an atrophic sample), and one individual with an SCI (referred to as severe atrophic sample). For detailed individual characteristics and the clinical history, see Tables 1 and 2. The muscle biopsies were obtained from the medial gastrocnemius muscle after local anesthesia (1% lidocaine) and an incision of the overlying skin. The biopsies were taken using a 5-mm Bergstrom needle (Stille, Stockholm, Sweden) with manual suction. The obtained muscle biopsies weighed around 80–100 mg. The present protocol was in accordance with the Helsinki Declaration and approved by the Regional Ethics Committee for the Capital Region of Copenhagen (protocol number H-2–2014-028).

**Table 1:** Participant characteristics.

	Age	Gender	Height (m)	Weight (kg)	BMI (kg/m^2^)	Ambulant
Control individual	32	Male	1.93	83	22.3	Yes
Stroke individual	38	Male	1.80	89	27.5	Yes
SCI individual	54	Male	1.84	95	28.1	No

**Table 2:** Clinical history.

	Injury	Spasticity	Medication	Other diagnosis	Smoking habits	Alcohol habits
Stroke individual	Hemiplegic due to stroke (≈ 3 years prior to experiment). Walks with walking aids (GMFCS:2)	Experiences some spasticity however not significant—the patient does not recieve any treatment towards spasticity	Simvastin, Asasantin retard	High Choleterol	Non smoker	Not daily
Spinal cord injury individual	Total paraplegic (≈ 21 years prior to experiment) due to a traumatic spinal cord injury (TH11/12 fracture)	Experiences some spasticity however not significant—the patient does not recieve any treatment towards spasticity	None	Non-insulin-dependent diabetes (NIDDM) for 14 years. The patient has flexion contractures in the calfes of both legs	Non smoker	Not daily

*GMFCS* gross motor function classification system.

### Sample preparation

The biopsies were embedded in Bouin's fluid and stored at 4° Celsius for 48 h. Then, the biopsies were washed with 98% Ethanol for 5 min six times. Subsequently, the biopsies were stored in 98% EtOH at 4° Celsius until imaging.

### Acquisition of data

The 3D imaging of the intact muscle sample was done at the TOMCAT beamline at the Swiss Light Source (proposal number: 20140425) using synchrotron X-ray computed tomography (SXCT). The central part of the biopsy was imaged with a cone beam at 25 keV energy using 1500 projections covering a 180° rotation. A filtered back-projection algorithm was used for extended tomographic reconstruction, resulting in an image of 2560 × 2560 × 2160 voxels. The voxel size was (330 nm)^3^, so the captured volume covered a field-of-view (FOV) of 844 × 844 × 773 μm^3^. A visual inspection revealed a poor image quality in the extended cylindrical periphery of the volume, due to the lack of full illumination of x-rays in this region. The volumes were down sampled (trilinear interpolation) by a factor of four, i.e., to a voxel size of 1.32 µm, to reduce the amount of data for the image processing and analysis. Indeed, some anatomical details were lost due to downsampling, but visually, the anatomical features to be quantified were still well preserved at the lower image resolution.

### Data processing

**Figure 1:**
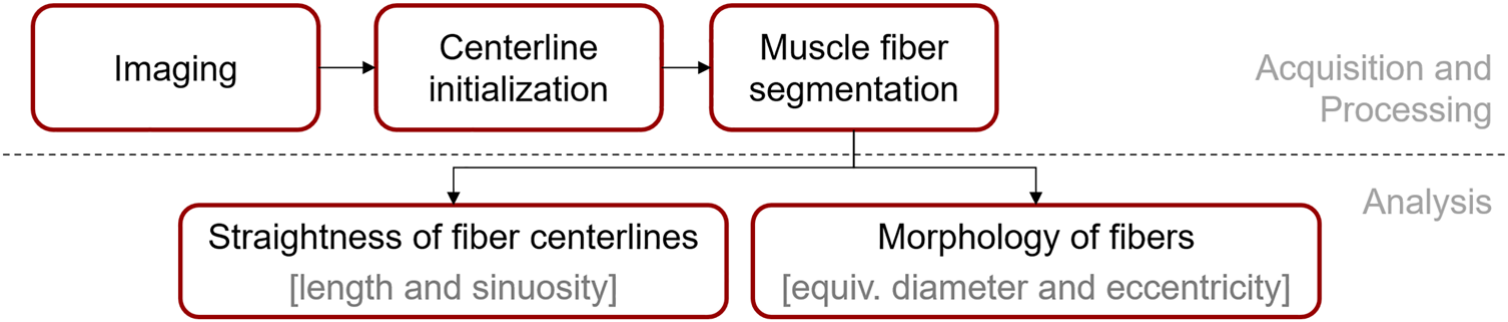
The processing pipeline for each sample.

The complete processing of the muscle fibers after the imaging required two steps as outlined in Fig. 1: first, the initial identification of each muscle fiber centerline, and secondly, a voxel segmentation of muscle fibers.

The first step was primarily a manual drawing process using ITK-Snap^25^. A thin mask was drawn in the central part of each muscle fiber, using the paintbrush tool to obtain an unbroken mask tracking the entire fiber length through the volume. This mask is the first guess of a centerline within a muscle fiber from which the centerline skeleton was extracted using an axis thinning algorithm^26^. The skeleton was processed to obtain the longest possible centerline without branches and equidistantly resampled with a chosen sectioning distance of 1.32 µm, equal to the voxel size of the image.

The second step was to produce a segmented mask of individual muscle fibers based on their centerline. From each point along the centerline, orthogonal cross-sectional sampling planes were defined. Voxel intensities were sampled along 60 spokes (with an angular resolution of 6°) projecting approximately 140 µm outwards from the centerline (with a sampling density of 3.2 µm). The spoke length was chosen such that the entire fiber was ensured to be encompassed, i.e., the maximum expected muscle fiber radius plus a safety margin. Since the muscle fibers appear with the brightest intensity in the images, a strong negative intensity gradient (2nd order central differences) along each spoke was used to model the outer border of a muscle fiber. To ensure a robust segmentation mask, we identified the surfaces of the muscle fibers by using a graph optimization problem as demonstrated in Jeppesen et al.^27^. The following geometrical assumptions of muscle fiber morphology were made to regularize the solution of the segmentation: the shape of individual muscle fibers was assumed to be a closed tubular structure, i.e., a cylinder with local deformations and trajectory variations along the centerline but without holes (except at the ends); In addition, there could be no overlap between neighbouring muscle fibers. Note, that the segmentation also provides a refinement of the centerline. The segmentation was implemented using Matlab (MathWorks, Massachusetts, USA).

### Data analysis

Each segmented muscle fiber was characterized based on the centerlines using the fiber length and straightness, and from the segmentation mask along the centerline, we derived metrics such as cross-sectional local thickness and roundness (Fig. 1). Morphology analysis is defined as the shape along individual muscle fibers whereas microarchitecture analysis is a muscle fiber population-based volumetric analysis here based on the fiber trajectories.

The length of each muscle fiber, *d*, was calculated as the summed Euclidean distance between each point of the centerline:

$$d={\sum }_{i=1}^{N-1}{\Vert {p}_{(i+1)}-{p}_{i}\Vert }_{2}^{2}$$

where *p* denotes the centerline as a set of *N* ordered points described with *(x, y, z)* coordinates.

Fiber straightness, *s*, was estimated by taking the ratio of *d* against the length of the straight line that connects the start and end point of each muscle fiber centerline, i.e.:

$$s=\frac{d}{{\Vert {p}_{N}-{p}_{1}\Vert }_{2}^{2}}$$

The sinuosity index has the range of [1 ∞], where a 1 describes a straight fiber. Fiber length and sinuosity are single scalar estimates per muscle fiber.

To characterize the local muscle fiber morphology (i.e., the size and shape of a muscle fiber), new 2D cross-sections of the segmentation masks were made. The cross-section was initiated from a point on the fiber centerline, *p_i*, and was oriented orthogonal to the local main direction of the fiber. Points along the centerline were sampled with a distance of 9.2 µm, which roughly represents a new sampling at every 7th slice of volume. This results in a sequence of binary masks for each muscle fiber which is quantified.

For each cross-section, the area, *A*, was estimated by fitting an ellipse to the perimeter to obtain the major and minor axis lengths, *a* and *b*. The ellipse area was used to calculate an equivalent circle diameter, *ø*, as a proxy for the local muscle fiber diameter as a size metric:

$${\o }_{i}=2\sqrt{\frac{A}{\pi }}$$

Further, the eccentricity, *e*, of the fitted ellipse provided a measure for the local fiber roundness as a shape metric:

$${the e}_{i}=\sqrt{1-\frac{{b}^{2}}{{a}^{2}}}$$

Eccentricity is an estimate between [0 1[, where 0 is perfectly circular, and 1 approaches a line.

We restricted the morphological analysis to the cylindrical volume within the FOV that was fully illuminated by x-rays during acquisition. If parts of a cross-sectional binary mask extend the cylindrical region of full illumination or hit the bottom or top of the sample, it was discarded from the analysis.

## Results

### Muscle fiber compositions vary with severeness of muscle atrophy

Muscle fiber segmentations revealed a large difference between the total number of muscle fibers for each category of muscle atrophy, as shown in Table 3. In the healthy control sample, the volume fraction was 74.7% (percentage of voxels classified as muscle tissue). In addition, the sample showed that all fibers were tightly packed. In comparison, the stroke sample (atrophic sample) contained 2.3 times more fibers, which were on average 1.5 times smaller in diameter but still tightly packed and making up a volume fraction of 70.2% (Table 3). The SCI sample (severe atrophic sample) contained 0.46 times fewer and loosely packed fibers and had a low volume fraction of only 35.3%.

**Table 3:** Sample characteristics.

Sample	Control sample	Stroke sample	SCI sample
Number of fibers	146	342	68
Total length [mm]	82.6	168.4	39.0
Fiber non-straigthness (median;IQR)	1.02; 0.02	1.06; 0.09	1.12; 0.11
Fiber thickness (median; IQR) [microns]	74.4; 10.4	50.2; 18.3	71.3; 28.8
Fiber non-roundness (median;IQR)	0.54; 0.17	0.57; 0.20	0.43; 0.20

### Longitudinal muscle morphology changes

**Figure 2:**
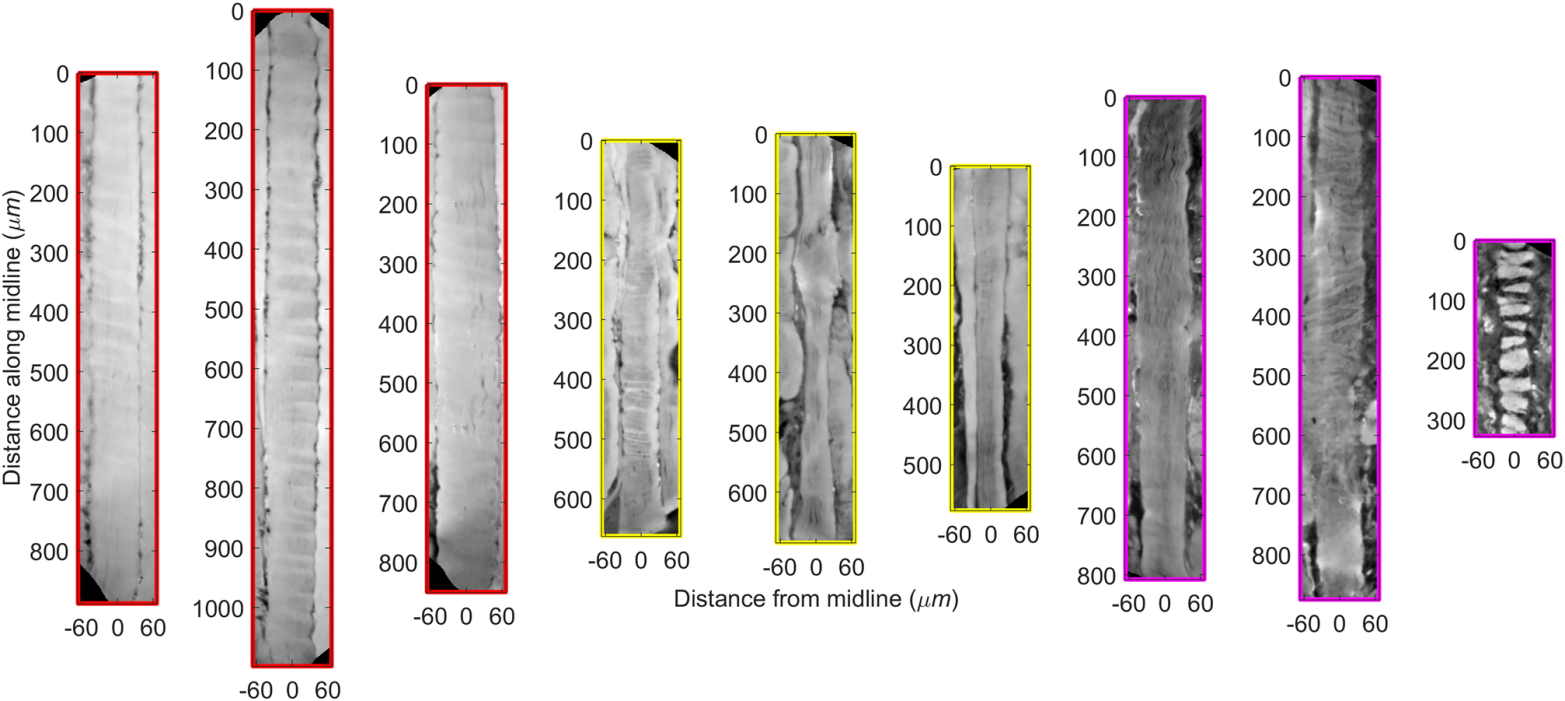
Muscle fiber appearance. Each plot shows the local image appearance around the centerline of selected muscle fibers which corresponds to an unfolded longitudinal cross-section. Three examples from each muscle sample respectively healthy (red), stroke (yellow), and spinal cord injury (purple). Image intensities were sampled in a small plane with a width of approximately 120 microns that follows the muscle fiber centerline, i.e., an unfolded longitudinal cross-section. The figure was created using MatLab software (v2020b) https://www.mathworks.com/products/new_products/release2020b.html.

**Figure 3:**
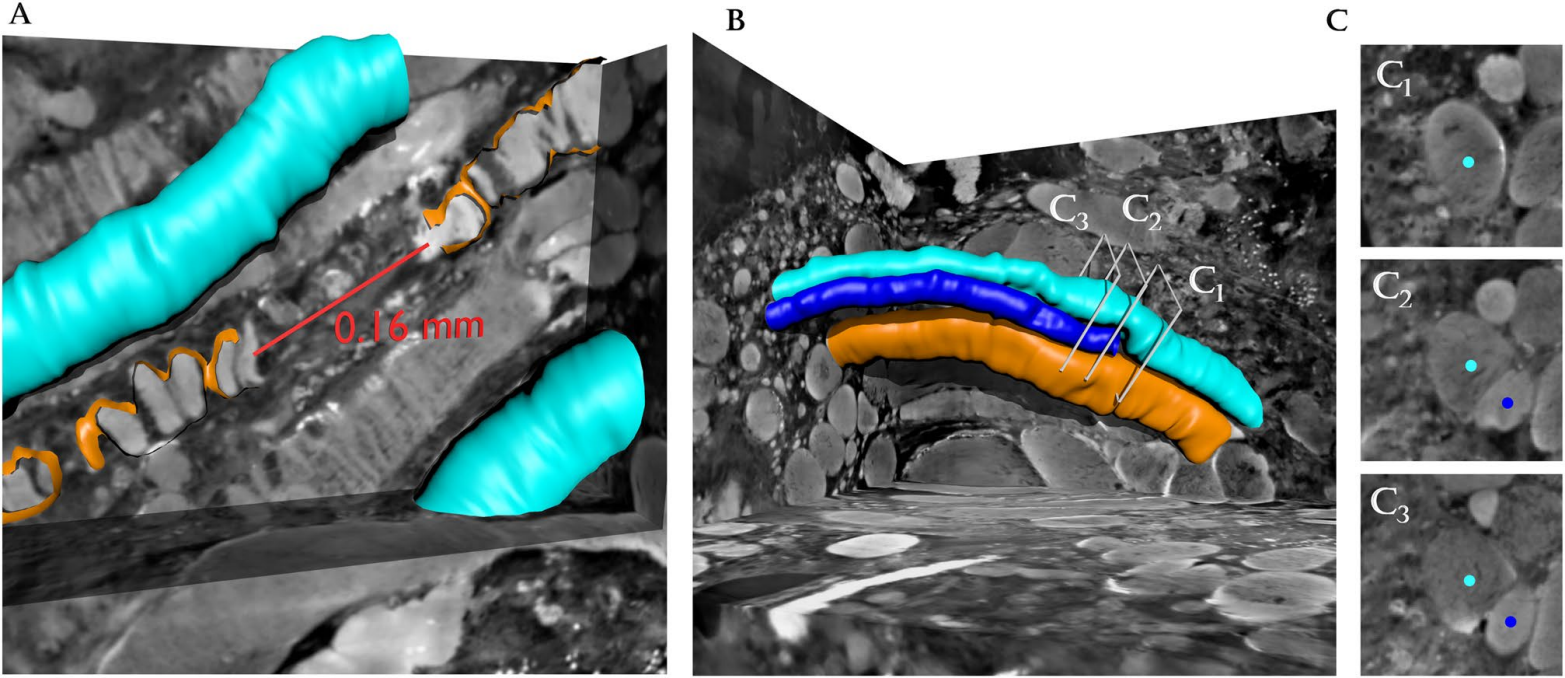
3D visualization of breaking and splitting fibers within the spinal cord injury sample. (**A**) Muscle fiber breaking: two heavily fractured muscle fibers were observed with a ‘gap’ between them of approximately 0.16 mm. Given the directionality of each segment and the neighboring muscle cells, it appears to be a single muscle fiber split into two. (**B**) Fiber splitting: the cyan fiber appears as one big muscle fiber until it splits into two separate neighboring cells (cyan and blue fibers). The disjoint appearance of the segmented objects is simply an artifact of our segmentation strategy, where each fiber is modeled as a tubular structure with no overlap with its neighbors. In reality, we would expect a smooth fusion from one fiber into two branches of fibers. (**C**) Three cross-sectional slices of the volumes around the merging point, supporting the visual appearance of the splitting. The figure was created using MatLab software (v2020b): https://www.mathworks.com/products/new_products/release2020b.html and Blender software (v2.78c): https://www.blender.org/download/releases/2-78/.

Figure 2 shows virtually unfolded longitudinal cross-sections of selected muscle fibers in 2D images, which highlight the qualitative differences in muscle morphology for the three selected examples of muscle atrophy. The healthy control sample showed muscle fibers that were smooth and homogenous in appearance and showed very little variation in diameter along the centerlines, as opposed to the fibers from the two other samples. Additionally, the SCI sample (severe atrophic sample) showed several examples of fibers that were broken into multiple disjoint segments (Fig. 3a), cavities or bubbles within the muscle (not shown), and even the appearance of fiber splitting (Fig. 3b,c).

### Muscle straightness and 3D organization changes

**Figure 4:**
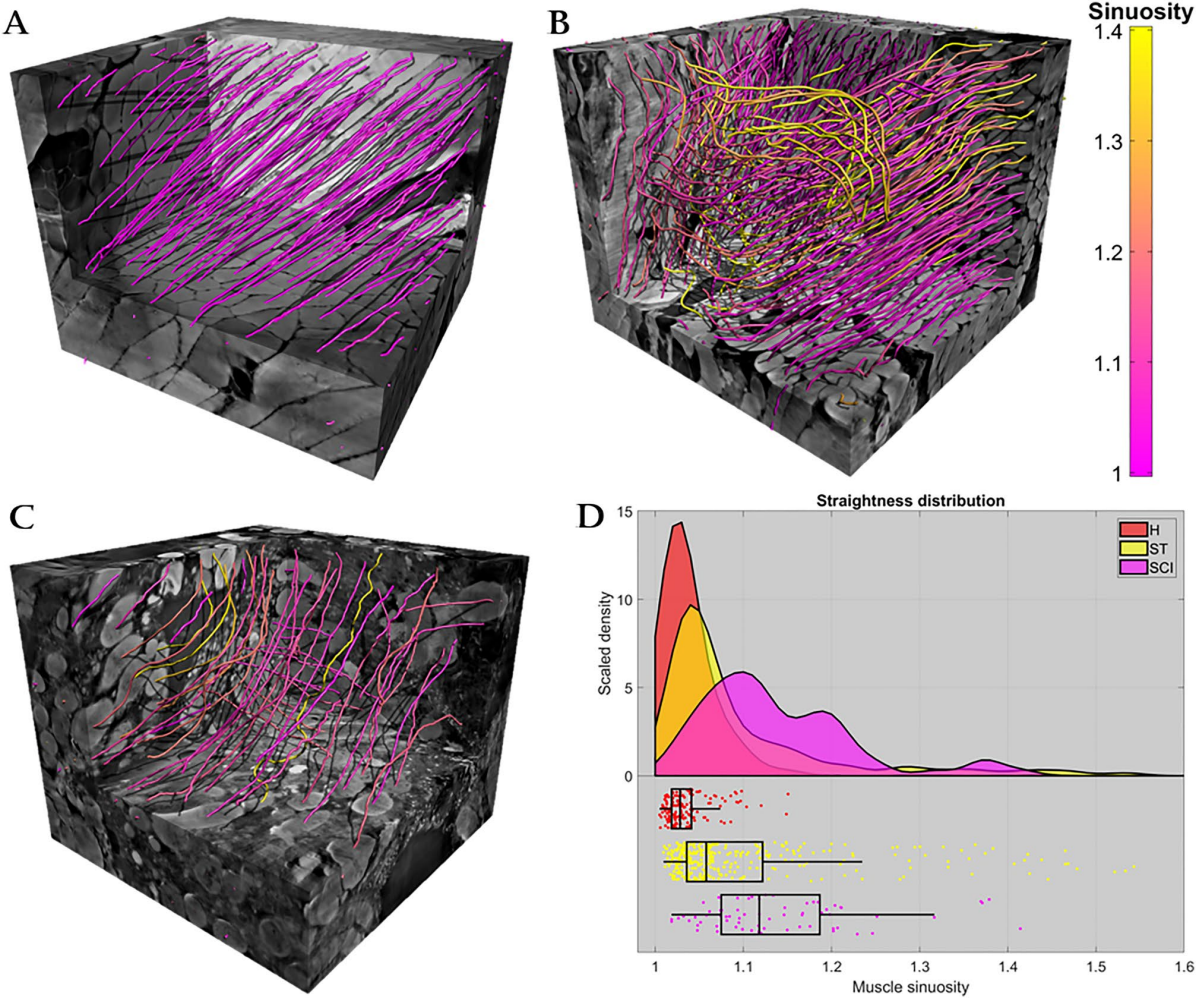
Analysis of muscle fiber straightness. (**A**–**C**) The detected centerlines within each of the three sample volumes, respectively in (**A**) the healthy control sample (H), in (**B**) the stroke sample (ST), and in (**C**) the spinal cord injury (SCI) sample. Each centerline is colored according to its sinuosity using the given color bar. (**D**) The statistics of centerline sinuosity for each sample. The distributions are a result of a kernel density estimation^53^. The boxplots indicate median value, 1st and 3rd quartile, and the whiskers are 1.5 IQR or truncated at extremum value. The figure was created using MatLab software (v2020b): https://www.mathworks.com/products/new_products/release2020b.html and Blender software (v2.78c): https://www.blender.org/download/releases/2-78/.

The relevant statistics for this analysis are summarized in Table 3 and Fig. 4d. The healthy control sample consisted of a population of straight muscle fibers (median s_H_ = 1.02) with a low variance (interquartile range (IQR) ‘straightness’ s_H_ = 0.02). Furthermore, a qualitative visualization of the healthy control sample microarchitecture revealed that the muscle fibers all shared a similar main direction (Fig. 4a).

In comparison to the healthy control sample, the stroke sample (atrophic sample) and the SCI sample (severe atrophic sample) showed decreasing muscle fiber straightness which increased with increasing severity of paralysis. Thus, the stroke sample (atrophic sample) had more non-straight muscle fibers with a median sinuosity index increasing to *s*_*ST*_ = 1.06. Interestingly, Fig. 4d shows that the stroke sample (atrophic sample) still had a large portion of straight fibers similar to the healthy control sample, and the most notable difference was the right-shifted and long-tailed distribution, also indicated by the increase in variance (IQR *s*_*ST*_ = 0.09). There were even a couple of particularly twisted muscle fibers whose sinuosity score exceeded a value of 2 (not shown in Fig. 4d). The microarchitecture of the muscle fibers in the stroke sample (atrophic sample) is shown in Fig. 4b by displaying the centerlines of the muscle fiber population. Note how some regions of the stroke sample (atrophic sample) showed deformed and disorganized muscle fibers, whereas some retained the characteristics of a healthy control sample, e.g. straight, well-organized muscle fibers.

In the SCI sample (severe atrophic sample), only a couple of fibers fell within the ‘normal’ straightness range of the healthy control sample shown in Fig. 4d. The median fiber sinuosity increased to *s*_*SCI*_ = 1.12 indicating further reduced muscle straightness compared to the healthy control sample and the stroke sample (atrophic sample). While the variance of sinuosity had increased (IQR *s*_*SCI*_ = 0.11) compared to the healthy control sample, it was similar to the stroke sample (atrophic sample). The microarchitecture in the SCI sample (severe atrophic sample) was qualitatively (Fig. 4c) highly disorganized and deformed, and it was hard to pinpoint a main direction for the sample compared to the other two samples.

A non-parametric Wilcoxon rank sum test was used to reject equality of sinuosity medians between both the healthy and the stroke sample, and the healthy against the SCI sample (both with p < 0.001)^28^. Similarly, a non-parametric Brown-Forsythe test was used to reject equality of sinuosity variance between the healthy and the two other samples (both with p < 0.001)^29^.

### Muscle fiber diameters and shape changes

**Figure 5:**
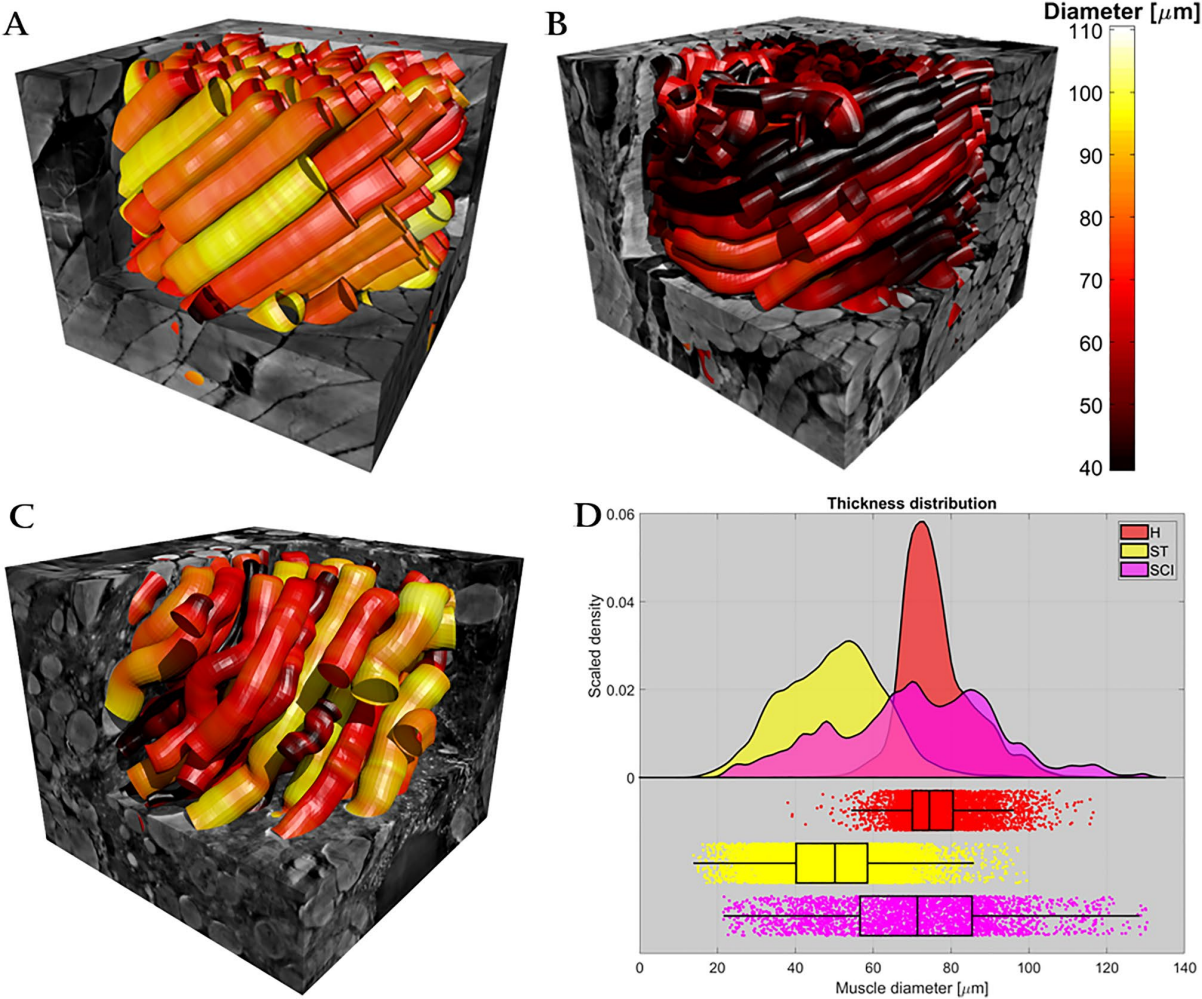
Analysis of local thickness. (**A**–**C**) The surface of segmented muscle cells within each of the three sample volumes, respectively in (**A**) the healthy control sample (H), in (**B**) the stroke sample (ST), and in (**C**) the spinal cord injury (SCI) sample. The segmentation surfaces are colored according to the local equivalent diameter using the given color bar. (**D**) The statistics of local fiber diameter for each sample. The distributions are a result of kernel density estimation. The boxplots indicate median value, 1^st^ and 3rd quartile, and the whiskers are 1.5 IQR or truncated at extremum value. The figure was created using MatLab software (v2020b): https://www.mathworks.com/products/new_products/release2020b.html and Blender software (v2.78c): https://www.blender.org/download/releases/2-78/.

**Figure 6:**
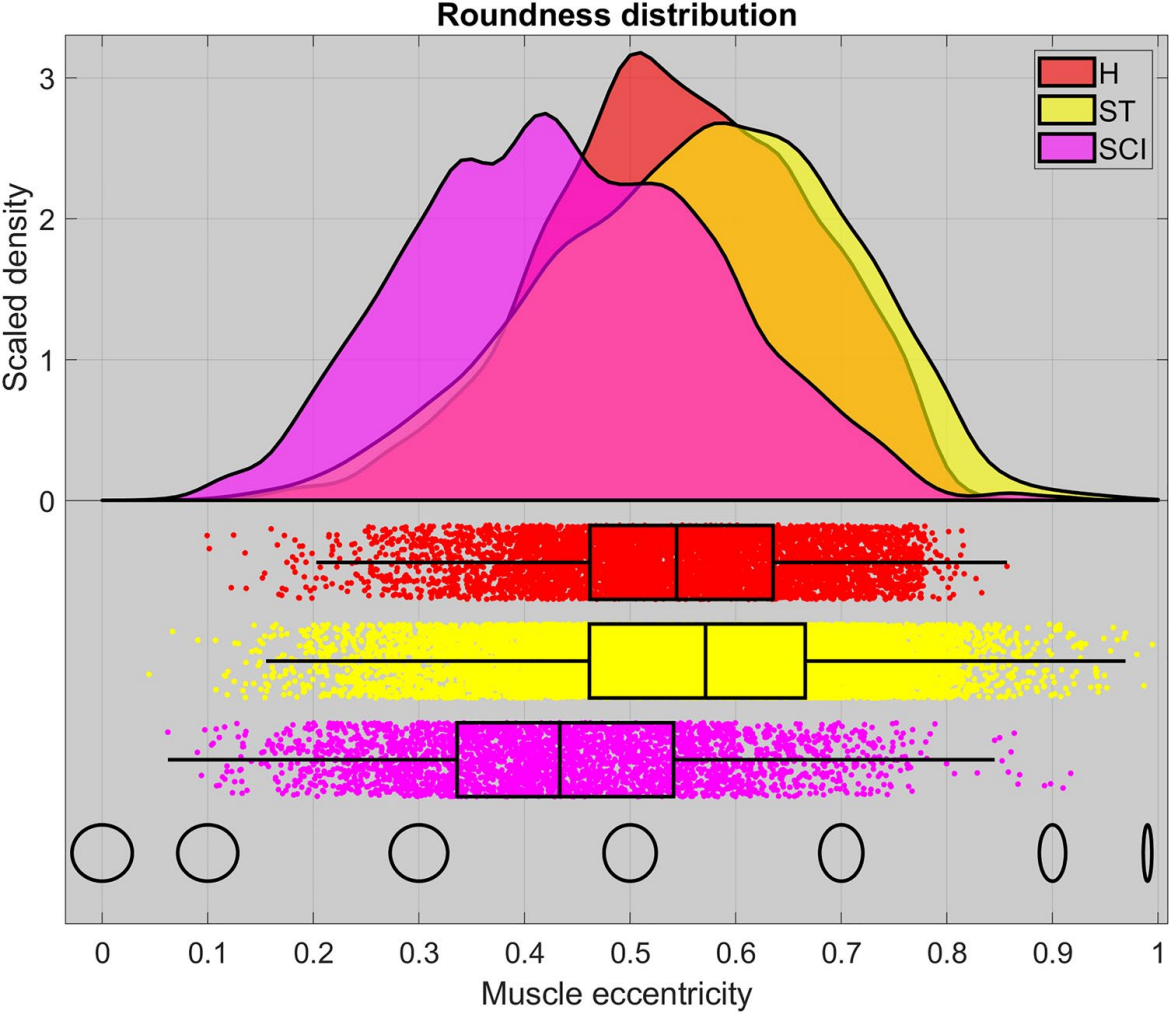
Analysis of roundness. The statistics of local fiber eccentricity for each sample. *H* healthy control, *ST* stroke, *SCI* spinal cord injury. The icons on the bottom row illustrate the nature of the eccentricity scale. The distributions are a result of kernel density estimation. The boxplots indicate median value, 1^st^ and 3^rd^ quartile, and the whiskers are 1.5 IQR or truncated at extremum value. The figure was created using MatLab software (v2020b): https://www.mathworks.com/products/new_products/ release2020b.html.

The relevant statistics for this analysis are summarized in Table 3, Figs. 5d and 6. Additionally, a consistent negative correlation (Pearson's linear coefficient) between diameter and shape (i.e., eccentricity) within each muscle fiber population was observed. However, the correlation is not particularly strong for any of the samples (respectively *r*_H_ = − 0.003, *r*_ST_ = − 0.23 and *r*_SCI_ = − 0.22).

The muscle fibers in the healthy control sample had a median equivalent diameter of *d*_*H*_ = 74.4 µm with relatively little variance both across the population (IQR *d*_*H*_ = 10.4 µm) and within each fiber. The latter is illustrated by the uniform coloring of each muscle fiber in Fig. 5a. None of the fibers were mathematically perfectly circular in their cross-sections and they had a median eccentricity of *e*_*H*_ = 0.54.

In the stroke sample (atrophic sample), the muscle fibers in Fig. 5b showed clear indications of atrophy with the median diameter dropping to *d*_*ST*_ = 50.2 µm (IQR *d*_*ST*_ = 18.3) but retaining a similar shape i.e. roundness of *e*_*ST*_ = 0.57 (Fig. 6). Interestingly, the SCI sample (severe atrophic sample) showed a large variation in fiber diameter (IQR *d*_*SCI*_ = 28.8 µm), but with a median size of *d*_*SCI*_ = 71.3, which was similar to that of the healthy control sample. The mixture of very small and large fibers in the SCI sample (severe atrophic sample) is illustrated in Fig. 5c. Furthermore, the figure demonstrates a large variation in the diameter of the individual muscle fibers. Surprisingly, the fibers of the SCI sample (severe atrophic sample) showed a tendency of being more circular in shape (Fig. 6), with a lower median eccentricity of *e*_*SCI*_ = 0.43.

A non-parametric Wilcoxon rank sum test was used to reject equality of medians in both diameter and eccentricity between both the healthy and the stroke sample, and the healthy against the SCI sample (all 4 cases with p < 0.001)^28^. Similarly, a non-parametric Brown-Forsythe test was used to reject equality of variance in both diameter and eccentricity between the healthy and the two other samples (all 4 cases with p < 0.001)^29^.

### Muscle fiber Buckling was observed in 3D but not in 2D segmentations

**Figure 7:**
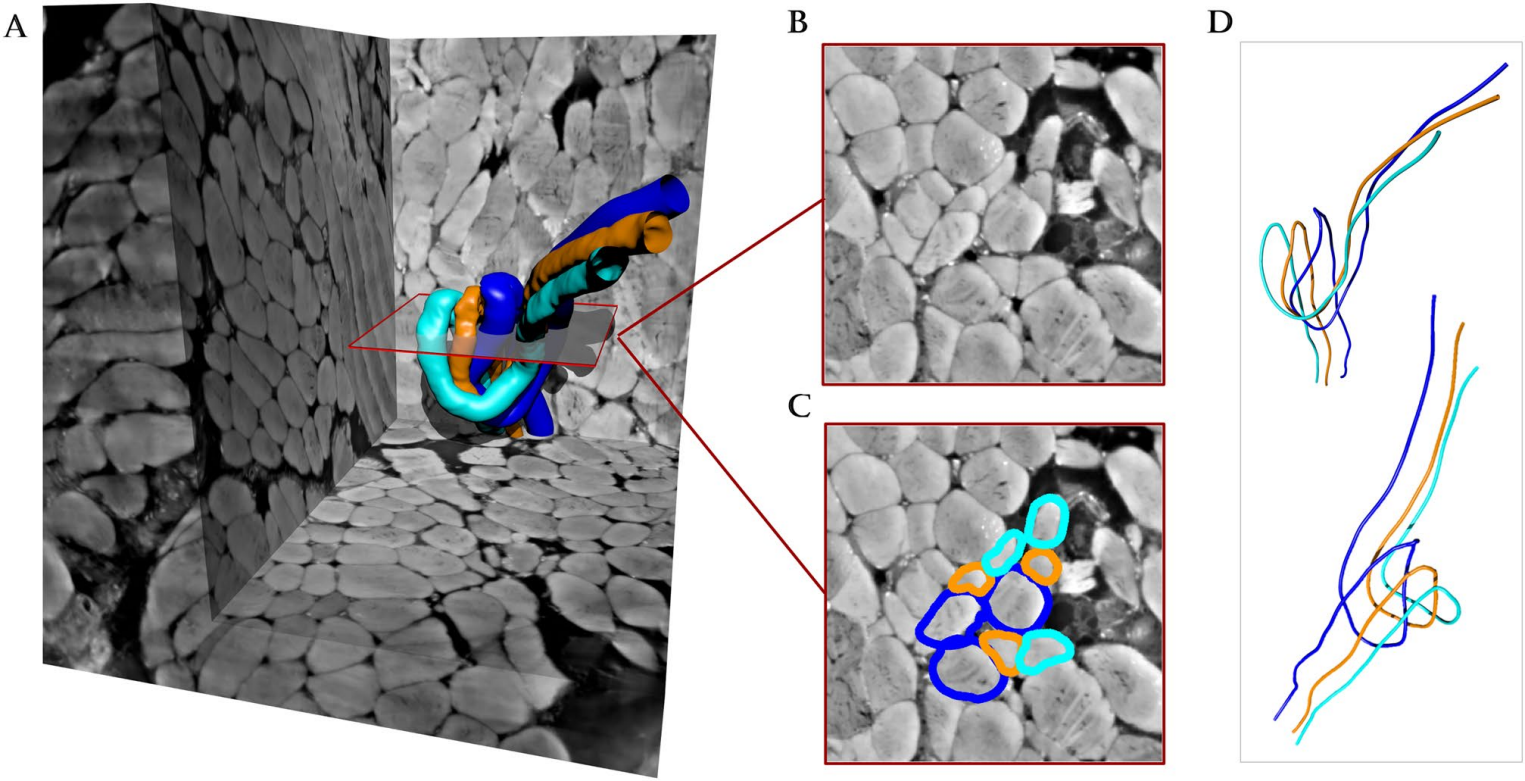
Buckling fiber visualization. (**A**) Rendering of segmented muscle fibers. (**B**) A small cut-out plane of the marked region. (**C**) Same region as B with borders of the segmentation overlaid. (**D**) Visualization of the muscle fiber centerlines from two different viewpoints. The figure was created using MatLab software (v2020b): https://www.mathworks.com/products/new_products/release2020b.html and Blender software (v2.78c): https://www.blender.org/download/releases/2-78/.

In the stroke sample (atrophic sample), the 3D trajectories of single muscle fiber centerlines revealed three examples of muscle fibers that were buckling, which are shown as orange, cyan and blue in Fig. 7. Interestingly, Fig. 7b,c showcase two cross-sectional slices (2D) from the 3D reconstructed muscle fiber as it would appear with classical histological analysis. In the 2D case, the bulking effect would incorrectly be classified as the trajectories of multiple closely neighboring muscle fibers.

## Discussion

The present study demonstrates that ultra-high image resolution synchrotron imaging of intact muscle biopsies (3–5 mm cubes) with its large field of view enables novel 3D insights into the morphology and organization of muscle fiber populations. The presented morphological metrics such as diameter and shape variations along fibers revealed clear changes between a healthy control muscle sample and two stages of muscle atrophy, i.e. atrophic (stroke) and severe atrophic (SCI). Furthermore, the analysis of muscle fiber trajectories in 3D enabled new insights into how the muscle fiber organization changes with atrophy which is not possible with conventional 2D histology.

The most striking observation was the buckling muscle fibers in the stroke sample (atrophic sample) observed in 3D but not in 2D sections (see Fig. 7). The buckling fibers were typically observed near one another, which may be interpreted as indicating localized focal points of muscle atrophy, from which further changes may subsequently radiate. Thus, the present result might be a visualization of the initiation of the muscle atrophy process in response to denervation. We propose the following theory as a theoretical explanation for this finding. We suggest, that when the muscle fibers decrease in size, they might lose their connection to their neighboring cells, causing a loss of lateral tension between the muscle fibers. When this tension is lost, it seems that the muscle fibers curl up and subsequently form these loops, resulting in muscle fiber buckling. This would cause a loss of function for the muscle fiber and subsequently cause a degradation of the muscle fiber. However, an empirical demonstration of this phenomenon remains to be seen in muscle tissue. Nevertheless, a previous study demonstrated a somewhat similar phenomenon of extracellular matrix and tenocyte buckling in tendon tissue of patients suffering from chronic tendinopathies using 3D reconstructions of transmission electron microscopy images^30^. The authors suggested that this phenomenon was caused by a local breakage of collagen fibrils caused by the absence of tensile load in the collagen fibrils in the tendon and thus resulting in contorted nuclei, misaligned cell bodies, and buckling of collagen fibrils, tenocytes, and the extracellular matrix^30^.

The 3D nature of the synchrotron data revealed another peculiar finding in the SCI sample (severe atrophic sample), the visualization of muscle fiber splitting from one fiber into two separate fiber segments (Fig. 3b). 2D histological and electromyographic studies have shown that fiber splitting is evident and a common degenerative change in different myopathies, including Duchennes muscular dystrophy and Polymyositis^31–33^. Furthermore, fiber splitting is also commonly seen in power athletes such as weightlifters after extensive exercise^34^. However, in these athletes, it is unknown whether the fiber splitting phenomena is caused by the real splitting of the fibers (hyperplasia) or by defective regeneration. A study by Eriksson et al. observed signs of degeneration and satellite cell activation in the muscle tissue of powerlifters, and the authors concluded that fiber splitting was caused by a defective regeneration of muscle fibers^35^ We propose that the present findings indicate that muscle splitting can be a natural process in the breakdown of a muscle cell during muscle atrophy as well as during muscle regeneration after intensive exercise.

The three samples that were included in the present study were 3–5 mm cubes of biopsies punctured from three human participants. The synchrotron setup enabled a large field of view. Since a large part of the sample (0.40 mm^3^ of tissue) was acquired in 0.33 micron, but analyzed in 1.32 micron isotropic image resolution, it enabled a 3D morphological analysis in an intact muscle fiber population. Indeed, quantification of certain morphological characteristics, including muscle fiber typing, fiber cross-sectional area assessment, and nuclei counting, have been reported previously^36–39^. Traditionally, these studies were made on 2D microscopy images^36^ which are usually only 5 µm thick or transmission electron microscopy samples that are approx. 1 mm thick and wide. Having a larger image volume, we presented several metrics to quantify muscle morphology and organization such as the number of fibers, the total length of the fibers, the non-straightness, cross-sectional shape, and the diameter (Table 3). However, these data have to be interpreted with caution since some of the numbers can be misleading, mainly due to the nature of puncturing tissue. For instance, the number of fibers represents unique fibers, but some of them can only be tracked for a short distance, e.g. if their trajectory is close to the FOV boundary.

### Organization of muscle fibers in 3D

Quantifying the straightness and morphology of 3D muscle fiber images appears sensitive to detecting different types of pathology. In the present study, it was observed that healthy muscle fibers ran in parallel and had a median sinuosity of 1.02. This is probably the highest degree of straightness that practically can be estimated when taking into account possible edge effectin the images and the uncertainty of the current analysis. The stroke sample (atrophic sample) showed an increased sinuosity (1.06), indicating lowered straightness, which agrees with the long muscle fibers being organized in a curved and crooked trajectory. On the other hand, the SCI sample (severe atrophic sample) showed fibers broken into multiple disjoint segments, indicating fiber splitting and formation of cavities and/or bubbles within muscle fibers. Nevertheless, the sinuosity index in the SCI sample (severe atrophic sample) increased further to a median value of 1.12, and therefore this straightness measure seems to be a decent indication of the microarchitectural order.

Similar levels of disorganization in the micro-architecture of muscle tissue have been observed in rats after chemical denervation using botulinum toxin A^40^. Together, these 3D observations and quantifications of various degrees of disorganization of muscle tissue might be a common feature of muscle atrophy. Thus, our 3D quantification of straightness and muscle fiber morphology analysis of 3D data can be used as biomarkers for muscle pathologies.

### Volumetric muscle fiber changes

The full segmentation of all muscle fibers within the image volumes allowed a quantification of the sample composition and measuring of the morphology. The results showed that muscle tissue from the healthy control sample had a high packing density and volume fraction of muscle fibers (74.7%), which means that the muscle fibers make up 74.7% of the volume of the sample. In the stroke sample (atrophic sample), the muscle fibers were still having a high packing density and volume fraction (70.2%), but the muscle fiber diameters were significantly smaller. In the SCI sample (severe atrophic sample) that was obtained from an individual with a spinal cord injury 21 years after the injury, only a few fibers were present, making up a volume fraction of 35.3%. These findings indicate that the stroke sample (atrophic sample) taken from an individual three years after the stroke suffers from muscle atrophy in the affected limb. However, ambulation might protect the muscles from dissipating unlike the wheelchair dependent individual 21 years after his SCI when the muscles appeared to disintegrate entirely. Thus, weight-bearing is most likely of great importance for skeletal muscle health and skeletal muscle function^41^. However, in a study using microgravity as a model for immobilization it has been shown, that even partial weight-bearing cannot protect the muscle from atrophy entirely^42^. In our study, this could explain why the individual who had a stroke still experiences some degree of muscle atrophy despite being ambulant. On the other hand, these findings indicate that using an individual who suffered a stroke and an individual with an SCI as a model for atrophy and severe atrophy, respectively, were successful.

The results of the present study further revealed that the diameter of the muscle fibers in the healthy control sample and the SCI sample (severe atrophic sample) was almost the same (Table 3: median: 74.4 and 71.3 µm. respectively). This was surprising given that the muscle fiber diameters of the stroke sample (atrophic sample) were noticeably smaller (median 51.2 µm). However, the SCI sample (severe atrophic sample) showed a considerable variation in fiber diameter, indicating both atrophy and hypertrophy. Since the volume fraction of muscle fibers was low in the SCI (severe atrophic sample), the remaining fibers had plenty of space to expand. Thus, we suggest that the reason for the large fiber diameters in the SCI sample (severe atrophic sample) might be caused by muscle fiber swelling.

Skeletal muscle fiber swelling is a mechanism that in previous studies has been associated with venous blood flow restriction (VBFR). VBFR is often applied together with resistance training to stimulate muscle hypertrophy^43–45^. The VBFR increases the extracellular pressure and thus increases the water flow into the cell^46^. This process increases the metabolites within the cell and subsequently leads to promoted hypertrophy^47^. However, in the present case of SCI injury, we suggest that the fiber swelling that has been observed might be caused by a shift in the water balance caused by other stimuli than VBFR. Which mechanism other than VBFR might cause muscle fiber swelling remains unclear.

While the present study is based on ex vivo experiments, the achieved insights concerning the 3D morphology of the muscle fiber morphology and organization can be useful in translating experiments into clinical settings. The signal of diffusion-weighted MRI (DWI) sequences is sensitive to the anatomy on a microscopic scale, even in-vivo^48^. However, as the resolution of the scanners is low, the fiber morphology and organization are indirect summary statistical estimates based on biophysical models to map fiber orientation distributions, volumetric, and diameter estiamtes^49^. The presented statistical distributions on morphology such as diameter and shape and organization related to non-straight trajectories, i.e., sinuosity can be the key to designing novel DWI protocols and biophysical models as was the case for axon diameter estimations in the brain^49,50^. Previous studies have used DTI to study muscle in vivo^49,51^, but the diffusion tensor model is only sensitive to the anisotropy of muscles and hence lacks specificity to morphology. More specific models of morphology applied to brain tissue can be translated to biophysical models of muscle microstructure^49^, but one must be aware of possible water exchange that influences the DWI-based metrics^51^.

### Limitations

The present study has several limitations. Firstly, we only studied muscle populations in three samples representing a healthy control individual, an individual that survived a stroke (atrophic sample), and an individual with an SCI (severe atrophic sample). Therefore, we cannot conclude whether the phenomena observed on the muscle population basis (fiber buckling, fiber splitting, and fiber swelling) are generally representative of groups of these CNS lesions.

We have only analyzed three muscle fiber populations, and there might be a variation between individuals due to various biological factors (age, gender, fitness level, nutritional state, etc.) that can lead to the muscle organization and morphology we observed. To confirm that our findings represent the different atrophic states as here seen in the stroke and SCI samples, further studies are needed with a larger sample size in each stage of atrophy. Nevertheless, the high number of quantified fibers allows us to estimate distributions of diameters and shapes of muscle fibers to describe muscle fingerprints of healthy vs. pathological muscle tissue. Currently, the distributions are created by slicing the muscle fibers with a distance of 9.2 microns. The choice of this parameter will affect both the estimated distributions and the statistical comparison tests. A more thorough investigation into the optimal choice of this parameter and its impact on the results is a task for future studies. However, data from more samples would allow comparisons between stroke and SCI as populations using the presented morphological metrics to verify our results. However, there might be a limitation in regard to how many samples can be scanned at an awarded beam time at a synchrotron facility. Newer micro-CT lab sources enable micro-meter image resolution with phase contrast, which might be easier to gain access to and allows for the collection of larger sample sizes^52^.

Another limitation lies in the sample preparation of the biopsy material. The biopsies were embedded in Bouin’s solution for 24 h and then moved into 96% ETOH until analysis. Unfortunately, the samples were not stained before the imaging, and therefore the contrast of the tissue (muscle fiber, intramuscular connective tissue, etc.) was weak. Any staining (such as osmium, iodine, etc.) would possibly have improved the contrast of the tissue and would have made the segmentation of the muscle fibers easier. Currently, we manually identify the centerline of each fiber. This is the most time-consuming step and the most significant bottleneck, preventing an upscaling of the experiment to include more samples.

## Conclusion

We used ultra-high resolution phase-contrast synchrotron imaging to image intact muscle biopsies to visualize and analyze individual muscle fibers in 3D. We segmented individual muscle fibers and presented metrics to analyze their morphology, i.e. diameter, shape, and the non-straightness of their trajectories. The methods revealed novel micro-architectural features of muscle tissue, including muscle fiber buckling and splitting. In addition, the present findings showed that partly denervated muscle tissue (muscle tissue after a stroke) had clear patterns of muscle atrophy in terms of changed morphology and organization. Total denervation, as seen after SCI, on the other hand caused muscle fiber swelling and necrosis. Finally, the present data revealed that tissue analyzed in 3D showed specific micro-architectural changes in the muscle tissue that were overlooked when analyzing muscle tissue in 2D.

## Data Availability

All analyzed data are available upon request (Corresponding author: jpingel@sund.ku.dk). All raw synchrotron files are publicly available at the Danish Research Centre for Magnetic Resonance, Copenhagen University Hospital Hvidovre and Amager, Denmark.
